# Inter-relation between diabetes mellitus and hypertension in terms of incidence and prediction in Saudi Arabia: a retrospective cohort study

**DOI:** 10.1186/s12889-024-19471-0

**Published:** 2024-07-22

**Authors:** Abdulhameed A. Alharbi, Alwaleed A. Alharbi, Sami Abdo Al-Dubai

**Affiliations:** Preventive Medicine Post Graduate Studies, Al Madinah Health Cluster, Al Madinah Al Munawarah, Ministry of Health, Al Madinah, Saudi Arabia

**Keywords:** Hypertension, Type 2 diabetes mellitus, Incidence density rate, Primary healthcare centers, Predictors, Saudi Arabia

## Abstract

**Background:**

Hypertension (HTN) and type 2 diabetes mellitus (T2DM) are interconnected metabolic disorders with escalating global incidence and prevalence. However, no longitudinal studies have specifically examined the incidence of HTN and T2DM in the same study population. This study aimed to elucidate the association between HTN and T2DM and ascertain their respective roles in the development of each other.

**Methods:**

This retrospective cohort study encompassed 809 Saudi patients from primary healthcare centers in Al Madinah Al Munawarah, Saudi Arabia. The sample was stratified into three cohorts: 226 patients with HTN but without T2DM, 274 patients with T2DM but without HTN, and 309 patients devoid of both T2DM and HTN. Over a retrospective follow-up period of approximately 5 years, incidence density rates (IDR) were computed for HTN in the T2DM cohort, T2DM in the HTN cohort, and both HTN and T2DM in the control cohort. Multiple logistic regression analysis was employed to identify predictors of HTN and T2DM.

**Results:**

The IDR of T2DM among patients with HTN stood at 73.9 (95% confidence interval [CI] 56, 92) per 1000 person-years, in contrast to 33.9 (95% CI 24, 44) per 1000 person-years in the control cohort (adjusted odds ratio [OR] = 7.1, 95%CI 3.55, 14.13). Conversely, the IDR of HTN among patients with type-2 T2DM was 55.9 (95% CI 42, 70) per 1000 person-years, while in the control cohort, it was 20.8 (95% CI 13, 28) per 1000 person-years (adjusted OR = 5.8, 95% CI 3.11, 11.09). Significant predictors of HTN in the logistic regression model encompassed age, smoking status, family history of HTN, T2DM status, and body mass index (BMI). Similarly, significant predictors of T2DM in the logistic regression model included age, sex, family history of T2DM, HTN, and BMI.

**Conclusion:**

This study unveils HTN and T2DM as mutually significant risk factors. The IDR of each condition in the presence of the other significantly exceeded that among individuals devoid of HTN or T2DM.

## Background

Hypertension (HTN) and type 2 diabetes mellitus (T2DM) are interrelated metabolic diseases with increasing incidence and prevalence worldwide [1]. They pose significant challenges to global public health as major preventable risk factors for cardiovascular disease and premature death [2]. In 2019, there were 463 million people with T2DM, which is expected to increase to 578 million by 2030 and 700 million by 2045 [3, 4]. Most people with T2DM live in low- and middle-income countries, which are expected to see the greatest increase over the next 19 years [5]. From 1990 to 2019, the global incidence of HTN increased twofold [6]. According to the World Health Organization (WHO), approximately 1.28 billion adults between the ages of 30 and 79 years are estimated to have HTN worldwide, with the majority (two-thirds) residing in low- and middle-income countries [7, 8].

HTN and T2DM tend to occur simultaneously and progress over time [1]. They result from metabolic syndrome [8, 9], developing sequentially in the same individual [10]. There is substantial evidence of an increased prevalence of HTN in patients with T2DM [10, 11]. The prevalence rate of HTN among T2DM is higher than that in age- and sex-matched patients without T2DM, ranging from 32 to 82% [10, 11]. Moreover, 50% of individuals with HTN have impaired glucose tolerance or T2DM [12].

In Saudi Arabia, HTN and T2DM are increasing at alarming rates [13]. According to recent data, 9.2% of Saudi adults ≥ 15 years old have HTN, increasing to 50% among individuals ≥ 65 years old [13]. However, Saudi Arabia is among the top 10 countries worldwide with the highest prevalence of T2DM, and it is projected to be among the top 5 countries with the highest prevalence of T2DM by 2030 [14]. In 2014, the crude prevalence of T2DM in Saudi Arabia was 13.4%, and this prevalence increases with age [14]. Despite the high prevalence of HTN and T2DM in Saudi Arabia, few studies have investigated their association.

Effectively managing HTN and T2DM poses numerous challenges, encompassing factors at the patient, provider, and system levels [15]. Epidemiology studies of these conditions are pivotal in clinical practice and public health, leading to a greater understanding of their impact [15]. As primary healthcare centers are the initial points of contact for individuals, families, and communities, it is crucial to adopt an integrated approach at this level to address the burden of HTN and T2DM and discern which condition can contribute more to the incidence of the other [15].

In medicine, incidence refers to the newly diagnosed cases of a disease or condition within a specific at-risk population over a specified timeframe [16]. Several studies have demonstrated an interrelationship between HTN and T2DM, and some have investigated HTN prevalence in patients with T2DM. However, no longitudinal studies have specifically examined the incidence of each condition in the same study population. This study aimed to examine the relationship between HTN and T2DM in terms of its incidence and prediction.

## Methods

### Study setting and population

This retrospective cohort study was conducted in 2023 at primary healthcare centers (PHCs) in Al Madinah Al Munawarah, Saudi Arabia. The inclusion criteria were adult patients aged > 40 years of both sexes, primarily diagnosed with either HTN or T2DM at baseline for exposed cases, and those without HTN or T2DM at baseline for non-exposed cases (control). We ensured that controls had normal blood pressure and were not prediabetics. The study excluded patients with a dual diagnosis of T2DM and HTN, pregnant women, patients diagnosed with type1 DM, patients with incomplete medical records, or those who discontinued follow-up.

To ensure a representative sample, we randomly selected 5 primary healthcare centers from Al Madinah Al Munawarah City from 40 PHCs. Convenience sampling was used to select participants who met the eligibility criteria. Accordingly, we recruited 226 patients with HTN and without T2DM, 274 patients with T2DM and without HTN at baseline, and 309 patients who did not have T2DM and HTN at baseline (the non-exposed or control group).

### Study instrument and procedure

Baseline data were extracted from the medical records of participants registered between 2010 and 2012. The patients’ periodic follow-ups for a subsequent period of 5 years were reviewed. Baseline and follow-up data were extracted using a customized checklist form encompassing demographic factors, including age at baseline, sex, marital status, education, body mass index (BMI), smoking, exercise, and family history (FH). Follow-up data included the mean of the last 10 systolic and diastolic blood pressure measurements for patients with HTN and the mean of the last 3 hemoglobin A1c (HbA1c) readings for patients with T2DM.

The diagnoses of HTN and T2DM were based on physician statements in the medical records according to the Saudi Hypertension Guidelines [17] and Saudi Diabetes Clinical Practice Guidelines [14]. BMI was determined using the patient’s weight and height at baseline, and it is classified according to the WHO categories: underweight (< 18.5 kg/m^2^), normal weight (18.5–24.9 kg/m^2^), overweight (25.0–29.9 kg/m^2^), or obese (≥ 30.0 kg/m^2^) [18]. In terms of glycemic control, an HbA1c level < 6.5% was considered an effective control, while an HbA1c level ≥ 6.5% was categorized as inadequate control, aligning with the American Diabetes Association guidelines [19]. The data collectors were trained in the data collection process, and the completeness of each dataset was verified during extraction.

### Ethical consideration

Ethical considerations were considered when collecting data, including obtaining ethical approval for conducting the research and ensuring the confidentiality of the names and personal information of patients. This study was approved by the Institutional Review Board (IRB)/Ethics Committees of Al Madinah Health Cluster, Ministry of Health (IRB2020-577). Written informed consent was waived by the Institutional Review Board (IRB)/Ethics Committees of Al Madinah Health Cluster, Ministry of Health because the study relies on secondary data and patient access is not possible.

### Statistical analysis

Statistical analysis was performed using Statistical Package for the Social Sciences (SPSS Inc., Chicago, IL, USA), version 22. For categorical variables, descriptive statistics were employed by calculating frequencies and percentages, whereas means and standard deviations were calculated for scale variables. The incidences of HTN and T2DM were estimated per 1000 person-years at risk. This was calculated by dividing the number of new cases diagnosed over the study period by the total follow-up time (per year) for people at risk of developing HTN, T2DM, or both during that period, multiplied by 1000. The backward LR technique was used to conduct a multiple binary logistic regression analysis to predict T2DM and HTN. Odds ratios (OR) and 95% confidence intervals (95%CI) were estimated to assess the strength of association between the outcome and variables in the model, with multicollinearity assessed within this model. Statistical significance was defined as *P* < 0.05.

## Results

### Sociodemographic and health-related characteristics of the participants

In the HTN group, the mean age (SD) was 62.4 (6.5) years, ranging from 45 to 70 years. More than half (57.5%) were male, and the majority were married (73.5%), had a primary education level (57.5%), and were non-smokers (70.8%). FH of HTN and T2DM were reported in 21.9% and 10.2%, respectively. The mean BMI was 30.2(4.9), with a range of 17.2 to 41.1. The mean (SD) follow-up period was 4.3 (0.6) years, ranging from 2.5 to 5 years (Tables 1 and 2).

**Table 1:** Sociodemographic characteristics of the participants

	Variable		HTN Group (n = 226)		T2DM Group (n = 274)		Control Group (n = 309)	
			N	%	N	%	N	%
Age		≤ 60	77	34.1	112	40.9	143	46.3
		> 60	149	65.9	162	59.1	166	53.7
Sex		Male	130	57.5	170	62.0	166	53.7
		Female	96	42.5	104	38.0	143	46.3
Marital status		Single	1	0.4	7	2.6	5	1.6
		Married	166	73.5	205	74.8	221	71.5
		Divorced	24	10.6	16	5.8	26	8.4
		Widowed	35	15.5	46	16.8	57	18.4
Education		Illiterate	3	1.3	15	5.5	13	4.1
		Primary	130	57.5	149	54.4	89	28.2
		Intermediate	45	19.9	43	15.7	82	25.9
		Secondary	17	7.5	18	6.6	59	18.7
		University	31	13.7	49	17.9	22	7.0
Smoking		Yes	66	29.2	82	29.9	93	30.1
		No	160	70.8	192	70.1	216	69.9
Exercise		Regular exercise	21	9.3	19	6.9	25	8.1
		Irregular exercise	71	31.4	97	35.4	106	34.3
		No	134	59.3	158	57.7	178	57.6

**Table 2:** Health-related characteristics of the participants

Variable		HTN Group (n = 226)		T2DM Group (n = 274)		Control Group (n = 309)	
		N	%	N	%	N	%
FH of HTN	No	168	78.1	232	84.7	237	81.7
	Yes	47	21.9	22	8.0	53	18.3
FH of T2DM	No	193	89.8	175	63.9	234	80.7
	Yes	22	10.2	79	28.8	56	19.3
Controlled HTN	Yes	107	48.4	-	-	-	-
	No	114	51.6	-	-	-	-
Controlled T2DM	Yes	-	-	3	1.1	-	-
	No	-	-	271	98.9	-	-
	**Mean (SD)**	**Minimum, Maximum**	**Mean (SD)**	**Minimum, maximum**	**Mean (SD)**	**Minimum, maximum**	
BMI	30.2 (4.9)	17.2, 41.1	30.8 (4.8)	16.5, 49.9	30.8 (4.3)	17.2, 49.9	
Follow-up period	4.3 (0.6)	2.5, 5	4.6 (0.5)	2.5, 5	4.7(0.4)	3.5, 5	

FH: Family history, BMI: Body mass index

In the T2DM group, the mean age (SD) was 60.4 (8.0) years, ranging from 43 to 70 years. The majority were males (62.0%), married (74.8%), had a primary education level (54.4%), and were non-smokers (70.1%). FH of HTN and T2DM were reported in 8% and 28.8%, respectively. The mean BMI was 30.8 (4.8), with a range of 16.5–49.9. The mean (SD) follow-up period was 4.6, (0.5) with a range of 2.5–5 years (Tables 1 and 2).

In the control group, the mean age (SD) was 60.8 (6.8) years, ranging from 41 to 70 years. Of the participants, 53.7% were males, 71.5% were married, 28.2% had a primary education level, and 69.9% were non-smokers. FH of HTN and T2DM were reported in 18.3% and 19.3%, respectively. The mean BMI was 30.8 (4.3), with a range of 17.2 and 49.9. The mean (SD) follow-up period was 4.7, (0.4) ranging from 2.5 to 5 years (Tables 1 and 2).

### Incidence of T2DM and HTN

In the HTN group, 65 patients developed T2DM (28.8%), corresponding to an incidence density rate of 73.9 (95% CI 56, 92) per 1000 person-years, while in the T2DM group, 63 patients developed HTN (23.0%), corresponding to an incidence density rate of 55.9 (95%CI 42, 70) per 1000 person-years. Conversely, in the control group, 46 patients developed T2DM (14.9%), corresponding to an incidence density rate of 33.9 (95%CI 24, 44) per 1000 person-years, and 29 patients developed HTN (9.4%), corresponding to an incidence density rate of 20.8 (95% CI 13, 28) per 1000 person-years (Tables 3 and 4).

**Table 3:** Incidence of T2DM among patients with HTN and controls

	New Cases of T2DM (%)	Follow-up per person-years	IDR Per 1000 person-years	95% CI of IDR
HTN population	65(28.8%)	879.8	73.9	56, 92
Controls	46 (14.9%)	1353.5	33.9	24, 44

IDR: incidence density rate

**Table 4:** Incidence of HTN among patients with T2DM and controls

	New Cases of HTN (%)	Follow-up per person-years	IDR per 1000 person-years	95% CI of IDR
T2DM population	63 (23.0%)	1127.0	55.9	42, 70
Controls	29 (9.4%)	1395.5	20.8	13, 28

IDR: incidence density rate

### Predictors of HTN in the multiple logistic regression analysis

The variables included in the multiple logistic regression analysis were age, sex, marital status, exercise, FH of T2DM, T2DM status, FH of HTN, BMI, education level, smoking status, and follow-up period. Factors that significantly predicted HTN in the logistic regression model were age, smoking status, FH of HTN, T2DM status, and BMI. Every 1-year increase in age increases the odds of HTN by 1.1 (95% CI 1.05, 1.14). Smokers were more likely to develop HTN than non-smokers (OR = 4.4, 95% CI 2.05, 9.60). Individuals with a positive FH of HTN were more likely to develop HTN compared to those with a negative history (OR = 25.7, 95%CI 11.72, 56.49). Those with T2DM were more likely to develop HTN compared to those without the disease (OR = 7.1, 95%CI 3.55, 14.13). Every one-unit increase in BMI increases the odds of developing HTN by 1.04 (OR = 1.1, 95%CI 1.04, 1.18). According to the Wald statistic values in the model, the most important predictor of HTN is a FH of HTN, followed by T2DM (Table 5).

**Table 5:** Predictors of HTN in the multiple logistic regression analysis

		B	Wald	OR	95% CI		P value
Age		0.085	16.189	1.1	1.05	1.14	< 0.001
Smoking	Yes	1.490	14.313	4.4	2.05	9.60	< 0.001
	No			1			
FH of HTN	Yes	3.248	65.484	25.7	11.72	56.49	< 0.001
	No			1			
T2DM	Yes	1.958	30.918	7.1	3.55	14.13	< 0.001
	No			1			
BMI		0.101	10.673	1.1	1.04	1.18	0.001

FH: Family history, BMI: Body mass index

### Predictors of T2DM in the multiple logistic regression analysis

The variables included in the multiple logistic regression analysis were age, sex, marital status, exercise, FH of T2DM, HTN status, FH of HTN, BMI, education level, smoking status, and follow-up period. The factors that significantly predicted T2DM in the logistic regression model were age, sex, FH of T2DM, HTN, and BMI. With every 1-year increase in age, the odds of T2DM increase by 1.1 (95%CI 1.01, 1.11). Males are more likely to develop T2DM compared to females (OR = 3.2, 95% CI 1.67, 6.21). Patients with a positive FH of T2DM had a higher chance of developing the disease (OR = 23.6, 95% CI 11.0, 50.17) than those without. Patients with HTN had a higher chance of developingT2DMthan those without the disease (OR = 5.8, 95% CI 3.11, 11.09). According to the Wald statistic values in the model, the most important predictor of T2DM is a FH of T2DM, followed by BMI and HTN (Table 6).

**Table 6:** Predictors of T2DM in the multiple logistic regression analysis

		B	Wald	OR	95% CI		P value
Age		0.059	5.920	1.1	1.01	1.11	0.015
Sex	Male	0.059	5.920	3.2	1.67	6.21	< 0.001
	Female			1			
FH of T2DM	Yes	3.160	67.330	23.6	11.0	50.17	< 0.001
	No			1			
HTN	Yes	1.764	28.949	5.8	3.11	11.09	< 0.001
	No			1			
BMI		0.262	40.234	1.3	1.23	1.41	< 0.001

FH: Family history, BMI: Body mass index

## Discussion

This study aimed to estimate the IDR of T2DM among patients with HTN and vice versa and to assess the relationship between T2DM and HTN as risk factors for each other. We found that the IDR of T2DM among patients with HTN was 73.9 per 1000 person-years (95% CI 56, 92), which closely aligns with the findings of a study conducted in Qatif, Saudi Arabia, where the IDR was 82.9 per 1000 person-years [20]. However, this was higher than the rate reported in China, where the IDR was 16.93 per 1000 person-years [21]. Conversely, the IDR of HTN among patients withT2DM was 55.9 per 1000 person-years (95% CI 42, 70), which aligns with another study conducted in Ethiopia reporting an IDR of HTN among patients with T2DM of 58.05 per 1000 person-years [22]. Nevertheless, this is lower than the rates reported in previous studies in Saudi Arabia and South Asia, where the IDR of HTN was 172.0 and 82.6 per 1000 person-years, respectively [23, 24]. Variations in the IDR could be attributed to differences in sociodemographic characteristics, healthcare services, and study settings.

The IDR of T2DM was 33.9 per 1000 person-years (95% CI 24, 44)in the control group, slightly lower than that reported by a study conducted in the USA (*among the Pima population*), where the IDR of T2DM was 38.9 per 1000 person-years [25]. However, it was significantly higher than the findings of other studies conducted in the USA (*among the South Asian population*) and China, where the IDR of T2DM was 16.1 and 13.4 per 1000 person-years, respectively [25, 26]. The IDR of HTN was 20.8 per 1000 person-years (95% CI, 13, 28), higher than the findings from studies conducted in Tabuk, Saudi Arabia, and Korea, where the IDR of HTN was 7.0 and 14.7 per 1000 person-years, respectively [27, 28]. However, this is lower than the finding of another study conducted in the USA, in which the IDR of HTN was 34 per 1000 person-years [29]. The findings of this study fall within the range of results observed in other studies conducted in different populations with different sociodemographic characteristics.

The relationship between HTN and T2DM in terms of their respective incidence is shown by comparing the IDR of each type and in the control group. This study revealed that the crude IDR of T2DM among patients with HTN was higher than the crude IDR of HTN among patients withT2DM (73.9 and 55.9 per 1000 person-years), suggesting that HTN may pose a higher risk factor for T2DM than vice versa. However, in comparison to the control group and after adjusting for confounders, we observed that patients with T2DM were more likely to develop HTN compared with those without the disease, whereas patients with HTN had a higher chance of developingT2DM than did those without the disease. Based on ORs, the risk of HTN in patients with T2DM was higher than the risk of T2DM in patients with HTN.

Diabetic kidney disease and cardiovascular complications can explain T2DM as a risk factor for HTN (30). In T2DM, hyperfiltration is linked to compromised renal autoregulation, leading to elevated arterial pressure [30]. Insulin resistance and DM can promote arterial stiffening, subsequently leading to HTN [31, 32]. Conversely, individuals with HTN have a higher progression rate of insulin resistance over time [33]. This is attributed to HTN-induced endothelial dysfunction, leading to adipose tissue inflammation and insulin resistance [34].

Age, smoking status, FH of HTN, T2DM status, and BMI significantly predicted HTN. The primary predictor of HTN in the model was a FH of HTN, followed by T2DM. HTN predictors are corroborated by the findings of other studies in Ethiopia and Saudi Arabia [22, 23]. The factors that significantly predicted T2DM were age, sex, FH of T2DM, HTN, and BMI. The primary predictor of T2DM in the model was a FH of T2DM, followed by BMI and HTN. T2DM predictors are supported by the findings of other studies in Saudi Arabia and China [20, 21].

The retrospective cohort design of this study allowed us to observe whether exposure preceded the outcome, potentially suggesting a causal relationship (although not as definitive as a prospective design). However, this study has a few limitations. A significant proportion of patients believe that primary healthcare centers are not ideal for monitoring and following up on their chronic conditions. Therefore, when a new health incident occurs, they autonomously decide to seek follow-up in a hospital or a larger medical center. This self-directed decision-making by patients may obscure the documentation of a new health incident, potentially leading to underestimated study findings. Therefore, follow-up studies should be conducted in hospitals or specialized medical centers. Furthermore, there are very few previously published studies specifically addressing the relationship between HTN and T2DM, which could serve as a point of comparison.

## Conclusions

HTN incidence in patients with T2DM is higher than T2DMincidence in patients with HTN. HTN and T2DM predicted each other after controlling for confounding factors. T2DM is a greater risk factor for HTN than HTN is for T2DM. Our findings encourage professionals to prevent HTN and T2DM by addressing modifiable risk factors such as obesity or being overweight, especially among those with non-modifiable risk factors such as positive FH and old age.

## Data Availability

The datasets used and/or analyzed during the current study are available from the corresponding author on reasonable request.

## Abbreviations

CI: Confidence Interval
BMI: Body Mass Index
T2DM: Type 2 Diabetes Mellitus
FH: Family history
HTN: Hypertension
IDR: Incidence Density Rate
OR: Odds Ratio
